# Pattern reversal chromatic VEPs like onsets, are unaffected by attentional demand

**DOI:** 10.1017/S0952523824000063

**Published:** 2024-12-16

**Authors:** Christabel Arthur, Osman B. Kavcar, Mackenzie V. Wise, Michael A. Crognale

**Affiliations:** Department of Psychology, University of Nevada, Reno, Reno, NV, USA

**Keywords:** Pattern reversal VEPs, attention, multiple object tracking, fMRI, visual pathways

## Abstract

Attention has been shown to modulate the visual evoked potential (VEP) recorded to reversing achromatic patterns. However, the chromatic onset VEP appears to be robust to attentional shifts. Functional magnetic resonance imaging (fMRI) responses to both chromatic and achromatic reversing patterns are also affected by attention. Resolution and comparison of these results is problematic due to differences in presentation mode, stimulus parameters, and the source of the response. Here, we report the results of experiments using comparable perceptual contrasts, pattern reversals, and a co-extensive and highly demanding multiple object tracking (MOT) task while exploring the effects of attentional modulation across both the chromatic (L − M) and (S − (L + M)) and the achromatic visual pathways. Our findings indicate that although achromatic VEPs are modulated by attention, chromatic VEPs are more robust to attentional modulation, even when using comparable stimulus presentation modes and in the presence of a highly demanding distractor task. In addition, we found that the majority of the modulation appears to be from a relative decrease in response due to the distractor task rather than a relative increase in response during heightened attention to the stimulus.

## Introduction

Reports on attentional modulation of neural responses in V1 have been varied. For instance, fMRI research has reported attentional modulation of the responses to both achromatic (Buracas and Boynton, 2007; Murray, 2008; Li et al., 2008) and chromatic stimuli (Song et al., 2011). VEP research has primarily reported attentional modulation of achromatic pattern-reversal but not chromatic onset stimuli (e.g. Heravian-Shandiz et al., 1992; Highsmith and Crognale, 2010; Wang and Wade, 2011). However, methodological and analytical differences make the comparison of the fMRI and VEP results difficult.

A review by Pashler et al. (2001) discussed the heterogeneity in stimulus properties that modulate attention. In fMRI research, attentional modulation of responses to visual stimuli has been reported using reversing patterns (Buracas and Boynton, 2007; Murray, 2008). Pattern-onset stimuli have been shown to be preferred for activating the chromatic pathways (Murray et al., 1987; Berninger et al., 1989; Kulikowski et al., 1989; Rabin et al., 1994; Highsmith and Crognale, 2010; Wang and Wade, 2011). These chromatic pattern-onset stimuli have, however, failed to show any significant modulation of attention in VEP recordings of early cortex. In a study conducted by Highsmith and Crognale (2010), attentional modulation of VEP amplitudes and latencies was compared when stimuli were presented as spatially contiguous and spatially separate using chromatic pattern onsets and achromatic pattern-reversal stimuli. They found no changes in both amplitude and latencies for chromatic pattern onsets but only attentional modulation of achromatic pattern-reversal stimuli. Wang and Wade (2011) similarly found no attentional modulation of the S-cone amplitude and phase responses to chromatic pattern onsets and reported modulation of attention to luminance amplitude and phase responses. In contrast, Di Russo et al. (2001) reported that attention increased the VEP amplitudes of both the luminance and chromatic reversal stimuli solely at high contrasts. When phases of the VEP were compared, only the luminance gratings were modulated by attention. The differences in temporal modality of presentation are particularly noteworthy given evidence that pattern reversals and pattern onsets preferentially modulate different populations of neurons (e.g. Strasburger et al., 1993). It remains unclear whether the different modes of presentation might account for the difference between the reports of attention modulation of achromatic and chromatic VEPs.

The spatial extent of attention is also a factor that affects attentional modulation of cortical activity. Behavioral studies have shown that the spatial location of a distractor affects performance (e.g., Beck and Lavie, 2005). While some studies have predominantly looked at selective attention to spatially separated distractor stimuli, other studies have targeted the VEP response to spatially co-extensive distractor stimuli (e.g., Heravian-Shandiz et al., 1992). Spatially co-extensive distractors are especially relevant to developmental or comparative studies wherein attention in the direction of the stimulus is often encouraged with attractive devices such as dangling toys or keys or even superimposing images or videos (e.g. Abramov et al., 1984).

Another factor that is important in evaluating attentional effects concerns the underlying source of the effect. Some studies have employed only two conditions, one in which the subject attended to the VEP stimulus and one in which they did not. (e.g. Di Russo and Spinelli, 1999). Others have employed an additional distractor task (Di Russo et al., 2001; Highsmith and Crognale, 2010). Consequently, it is not clear if attending to a stimulus increases the response to that stimulus, as has been claimed for the BOLD response of fMRI (Boynton, 2009), if a distractor task decreases the evoked response (Highsmith and Crognale, 2010), or if in fact both effects are present.

In addition to the above differences in experimental conditions, distractor task difficulty has also varied across prior studies. Some distractor tasks may not have been particularly demanding, while others may have required much attention (Meyerhoff et al., 2017). It is possible that a failure to observe attentional modulation in some cases was a result of insufficient attentional demand.

In our current study, we sought to answer the following questions:

- Are VEP responses to chromatic stimuli still robust to attentional modulation if the stimulus is reversed instead of presented as an onset? In other words, are the observed differences due to mode of presentation rather than differences in the chromatic and achromatic pathways?
- Are chromatic VEPs still unaffected by attentional modulation when using a highly demanding and co-extensive distractor task?
- Is attentional modulation of the VEP driven by changes in attention to the stimulus, or are they modulated by the presence or absence of a distractor task, or both?

To answer these questions, we employed reversing patterns with comparable perceptual contrasts and a co-extensive and highly demanding distractor task. We also included conditions that separately modulated attention to the stimulus and to the distractor.

## Methods

### Participants

Participants were 13 adults (seven males and six females) with a mean age of 29 years. All the participants had corrected to normal vision (two participants wore non-tinted glasses, and one wore non-tinted contact lenses) and were screened for normal color vision using the Ishihara 38-plate Test. Participants provided written informed consent. The study was approved by the Institutional Review Board of the University of Nevada, Reno.

### Procedure

To test whether or not differences between attentional modulation of chromatic and achromatic responses are due to differences in mode of presentation, we recorded the VEP to chromatic and achromatic reversing sinusoidal grating patterns that were roughly equivalent in perceived contrast. We used suprathreshold contrast matching to ensure that the perceptual contrasts are roughly equivalent and below the point of saturation (Rabin et al., 1994; Crognale et al., 1997; Switkes and Crognale, 1999). We also used data from prior experiments (Prescott et al., 2022; Wise et al., 2022) in which we obtained contrast response functions to ensure that responses were well below saturation. To confirm that prior failures to observe attentional modulation of chromatic responses were not due to the employment of an ineffective distractor task, we spatially superimposed a motion object tracking (MOT) task that was behaviorally demonstrated to be highly demanding. In order to separate response enhancement due to attention to the stimulus from the attenuation of the response due to attention to the distractor, we recorded the VEP while subjects were asked to either I) perform the MOT task, II) attend to the VEP stimulus pattern and detect a momentary decrease in contrast, or III) just stare at the center of the screen (no-task).

### VEP stimuli

**Figure 1.**
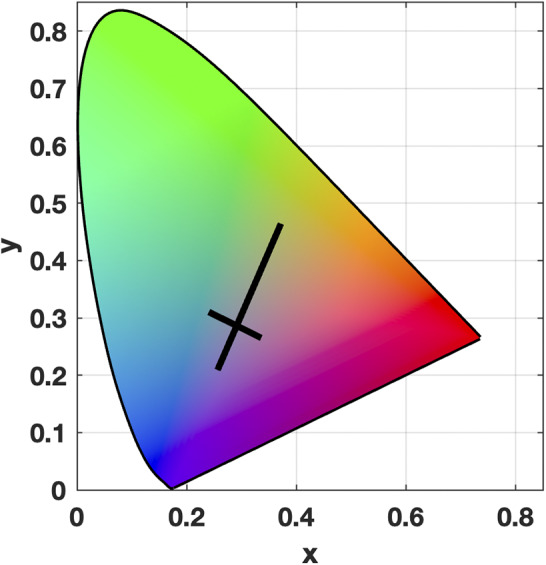
Pattern reversal stimuli in CIE color space. Solid lines show the extent of chromatic modulation along the two MDBKL cardinal chromatic axes.

VEP stimuli were generated using MATLAB (Mathworks, USA) and Psychtoolbox. We employed a vertical, sinusoidal pattern with a spatial frequency of one cycle per degree and a mean luminance of 67 cd m-^2^. The pattern was reversed in a square-wave fashion four times per second. Pattern colors fell along the cardinal axes of MBDKL color space (MacLeod and Boynton, 1979; Derrington et al., 1984). Participants first performed a minimum motion task to determine individual equiluminance settings along the (L-M) and S axes. The minimum motion task involved exchanging a low-contrast, achromatic Gabor patch with a chromatic, two-colored patch in spatial and temporal quadrature (Anstis and Cavanagh, 1983). The patches were oriented horizontally, so when the two colors of the chromatic patch differed in luminance, there was a luminance component that appeared to drift upwards or downwards depending on the phase of the brighter and dimmer colors. Participants used keyboard button presses to change the relative luminance of the two colors in the chromatic grating, causing the drifting grating to change directions when the luminance difference in the two colors was reversed. Participants adjusted the relative luminance of the colors until the patch appeared to contain little to no movement and only appeared to flicker. This setting was taken as the individual equiluminant point. Achromatic stimulus contrast (Michelson) was 0.18. The CIE coordinates for the endpoints of the axes were: +S; x = 0.256, y = 0.209; −S: x = 0.372, y = 0.464; +L−M; x = 0.3363, y = 0.2650; −L + M: x = 0.2403, y = 0.3104. (Figure 1). The stimulus monitor was calibrated using a PR-650 Spectra scan spectral radiometer (Photo Research Inc., Chatsworth, CA, USA). The VEP stimuli subtended a width of 53.5 degrees by a height of 32 degrees of visual angle.

### VEP recording

VEPs were recorded using Grass gold electrodes (Grass Technologies, West Warwick, RI, USA) and a Grass IPS 600 bio amplifier, digitized, and input to a PC using a National Instruments IO Board (National Instruments, Newbury, Berks, UK). A gain of 10,000 Hz was applied to the signal. The filter was set to a half-amplitude low cutoff of 0.3 Hz and a half-amplitude high cutoff of 100 Hz. No notch filtering was applied. Based on the international 10 – 20 system (Odom et al., 2016), the active electrode was positioned over the occipital lobe (Oz), with reference at the vertex (CZ) and ground on the forehead (FpZ). The impedance of the electrodes was kept below 10 kΩ (at 30 Hz). The electrodes were applied using Nuprep preparation jelly and Ten20 conductive paste (Weaver and Company, Aurora, CO, US). VEPs were recorded in six-second epochs, ten times, for a total of 60 seconds of recording per condition for each subject. Participants were positioned 57 cm from the screen.

#### Distractor task

**Figure 2.**
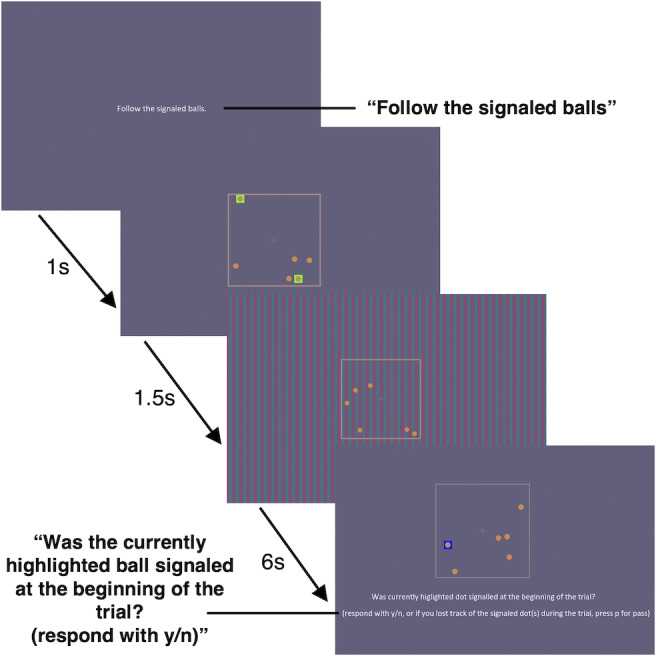
MOT distractor task sequence.

The Multiple Object Tracking Task (MOT) paradigm was adapted from Pylyshyn and Storm (1988). In this task, subjects are presented with an array of objects (typically filled circles), wherein a subset of the objects is temporarily indicated to be the “targets.” After the target indicators disappear, the objects in the array begin to move around in random directions for the trial duration. At the end of the trial, the objects stop moving, and the subject indicates which of the objects were the preselected targets. MOT performance tends to decline with an increasing number of targets, distractors, and trial duration (Meyerhoff et al., 2017). Since it is possible that attentional effects for the chromatic VEP might be revealed with more difficult distractor tasks, we performed a pilot experiment wherein the number of tracked balls in the MOT task was varied. We subsequently chose to use a 2-target tracking task and a 6-second trial duration, which had proved highly demanding and produced an average performance of 61% correct. Participants were presented with an array of six balls, each subtending 0.5 degrees, that moved randomly within a square, subtending 8 degrees by 8 degrees in the center of the stimulus screen. Two of the balls were highlighted at the start of the trial, and participants were asked to track those two balls as they moved around for 6 seconds while fixating on a small, central cross. At the end of the MOT trial, one of the six balls was highlighted, and participants were cued to respond yes or no to whether the ball highlighted at the end was one of the two balls highlighted at the beginning of the trial (Figure 2). Consequently, chance performance on the task was 33% correct.

#### Pattern attention task

In the pattern attention task, participants were instructed to fixate the cross but attend to the reversing pattern while ignoring the moving balls. To ensure an attentive state, for half of the trials, there was a momentary decrease in contrast to the grating during one of the reversals, and participants were instructed to respond to a prompt after the trial as to whether or not a contrast fluctuation occurred during the trial.

#### Stare condition

We included a condition wherein participants were instructed to just fixate the cross in the middle of the screen. This condition allowed us to determine if there were additive effects for a distractor task (that is does attending to a distractor produce a greater loss of the VEP response than just not attending to the VEP stimulus).

#### Control condition

We included a control condition wherein the VEP pattern stimuli were absent (grey background) during the MOT task and the stare task. This served to ensure that intrinsic activity (alpha) at the stimulus frequency and its harmonics or other artifacts from the presence of the distractor task are not contributing to the results. Finally, we recorded VEPs with only the pattern reversal stimulus present (no superimposed MOT task). This served as a relatively unbiased base for normalization of amplitudes, as these often vary enormously across participants as epiphenomena (skull thickness and skin impedance).

## Results

**Figure 3.**
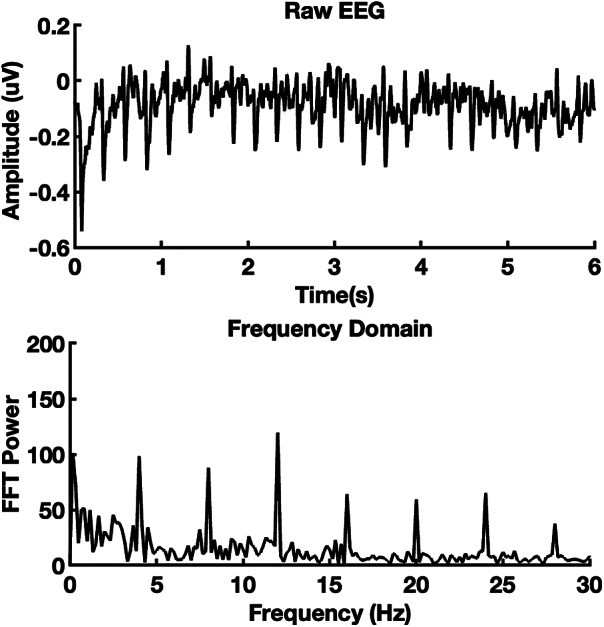
An example of EEG and Fast Fourier Transform from one subject. The 4 Hz and harmonic responses are evident as peaks in the frequency domain.

**Figure 4.**
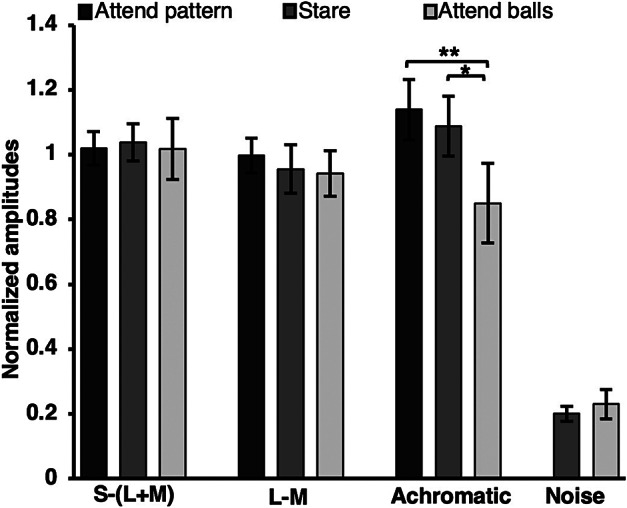
Response amplitudes across all conditions. Significant attentional modulation was observed only in the achromatic condition (indicated). Error bars = one standard error of the mean (SEM).

To quantify the VEP response, we used a MATLAB program (Mathworks, USA) to average the 10 six-second epochs of data collected in each of the 14 experimental conditions. Each subject’s average data was transformed from the time domain to the frequency domain using a fast Fourier transform (FFT) with a sampling rate of 1000 Hz and a FFT resolution of 0.167 Hz (Figure 3). Summed amplitudes of the first six harmonics of the reversal frequency (4 Hz, 8 Hz, 12 Hz, 16 Hz, 20 Hz, and 24 Hz) provided an overall response magnitude for each condition for each subject. Since amplitudes across individuals were highly variable (min = 13.86 μV, max = 524.96 μV), as is typical with EEG, we normalized the results for each subject by dividing VEP amplitudes of each condition by the amplitude produced by the pattern reversal grating alone, resulting in a response index. As expected, the control conditions (without VEP stimulus) elicited the lowest amplitudes and were taken as a measure of the “noise.” Also as expected, attention to the MOT task had no significant effect on the noise at the stimulus frequency and its harmonics (Figure 4).

We performed two-tailed paired T-tests across all conditions using a Bonferroni correction and found significance only for the achromatic responses. Specifically, we found a significant effect (*p* = 0.0002) when shifting attention from the pattern (mean = 1.14; SD = 0.34) to the distractor task (mean = 0.85; SD = 0.44). Interestingly, we found a significant decrease (*p* = 0.0050) when attending to the distractor compared to the no-attentional task (mean = 1.09; SD = 0.33). but no significant increase in response when attending to the pattern compared to the no-task condition (Figure 4). None of the attentional manipulations for the chromatic conditions produced significant amplitude changes. Reanalysis of the data, averaging over 1 second bins instead of 6 second bins, produced nearly identical results.

## Discussion

In this study, we looked at the effects of attentional modulation using pattern reversal chromatic and achromatic stimuli and a co-extensive and highly demanding distractor task. Chromatic VEP responses were found to be robust to attentional modulation when the stimuli were reversed, indicating that the previously reported lack of attentional modulation of the chromatic onset VEP was not due to the onset mode of presentation of the stimuli. It could also be argued that reversing patterns favor detection by transient pathways and that perhaps the reversing pattern response is not actually a “chromatic” response. However, our results demonstrate that the achromatic and chromatic pattern reversals must be preferentially stimulating different neural populations since attentional modulation affects them differently.

We also show that for achromatic responses, the attenuating effect of the distractor was larger in magnitude than the enhancement effect from specifically attending to the VEP stimulus. Our results further indicate that the chromatic pathway remains robust to modulation by attention even in the presence of a highly demanding, co-extensive distractor task.

The findings observed in this study reinforce those of previous studies that have failed to find attentional modulation of the chromatic pathways (Highsmith and Crognale, 2010; Wang and Wade, 2011). One plausible explanation for the lack of attention in the chromatic pathway is differential processing of the attentional mechanisms between the achromatic and chromatic pathways by cortical gain control mechanisms, as suggested by Di Russo and Spinelli (1999) and supported by Di Russo et al. (2001), Highsmith and Crognale (2010), and Wang and Wade (2011). However, fMRI evidence of attentional modulation of chromatic information processing in V1 has been reported previously (e.g. Song et al., 2011), even in the presumed presence of differences in gain mechanisms. It should also be noted that this imaging evidence was apparent only when applying multi-voxel pattern classifiers to differentiate orientation from color information and not in the summed neural response.

Our data suggest that attentional modulation of the achromatic VEP seems to be largely influenced by the presence of a distractor task. We found that attending to a distractor produces a greater loss of the achromatic VEP response than just not attending to the VEP stimulus, which is consistent with reports from Highsmith and Crognale (2010) demonstrating that the distractor task decreases the evoked response. However, the lack of enhancement of the response when attending to the stimulus differs markedly from evidence that the BOLD response of fMRI is typically enhanced when attending to a stimulus (Li et al., 2008; Boynton, 2009). Our stare condition and grating condition might be thought to have increased alpha, which could affect the attentional modulation of the pathways. However, alpha effects should be minimal since the conditions are randomized every 6 seconds. To check for alpha coherence effects, we reanalyzed the data using 1-second bins. The results were unchanged from those using 6-second bins, confirming that the bin size in the analysis does not appreciably alter the results. More importantly, since alpha intrusion is likely non-selective for pathways, it should not affect our conclusions supporting differences in attentional modulation between the chromatic and achromatic pathways.

The discrepancies between VEP and fMRI results may also be due to the physiological differences in the source of the signals (Lauritzen et al., 2010). For example, it is possible that the BOLD response of fMRI indirectly reflects local neural activity with feedback from higher cortical areas, while VEPs comprise potentials derived from pyramidal cell feed-forward activity. Consequently, the VEP may reveal the effects of attentional feedback mechanisms less efficiently than does the BOLD signal. The noted delay in attentional feedback mechanisms is another possible reason why fMRI appears to better reflect the effects of attention on perception (reviewed by Pessoa et al., 2003). Examination of attentional effects on VEP components with longer latencies was not possible with our current paradigm.

We utilized a two-ball, spatially co-extensive MOT task, which has been proven to produce significant behavioral effects (Beck and Lavie, 2005), and found attenuation of the achromatic response amplitudes. We did not observe attentional effects for either chromatic axis. This observation is therefore not likely due to insufficient attentional demand. The results are consistent with the study by Highsmith and Crognale (2010), where the distractor tasks were presented as both spatially co-extensive and spatially separate. However, Di Russo et al. (2001) found modulation of VEP amplitudes for both luminance and chromatic stimuli in the presence of spatially separate distractors.

VEPs are widely used to assess the integrity of the visual pathways. This is a particularly useful application for populations that are non-verbal or difficult to assess behaviorally (e.g. infants). Our findings provide support for prior conclusions that while avoiding active distraction is likely important, ensuring an “attentive state” is not always necessary when recording chromatic VEPs. Apparently, merely monitoring gaze direction is sufficient for chromatic VEP recording and diagnosis of vision disorders.
